# Life cycle inventory data for banana-fiber-based biocomposite lids

**DOI:** 10.1016/j.dib.2020.105605

**Published:** 2020-04-22

**Authors:** L. Joana Rodríguez, Serena Fabbri, Carlos E. Orrego, Mikołaj Owsianiak

**Affiliations:** a Facultad de Ingeniería y Arquitectura, Departamento de Ingeniería Industrial, Universidad Nacional de Colombia Sede Manizales, Bloque Q, 170003, Manizales, Colombia; b Quantitative Sustainability Assessment Group, Division for Sustainability, Department of Technology, Management and Economics, Technical University of Denmark, Produktionstorvet, Building 424, DK-2800 Kgs. Lyngby, Denmark; c Instituto de Biotecnología y Agroindustria, Departamento de Física y Química, Universidad Nacional de Colombia Sede Manizales, Bloque T, 170003, Manizales, Colombia

**Keywords:** Life cycle assessment, Banana fiber, Coffee jar lid, Biocomposite, Poly (lactic acid), High density polyethylene, Life cycle inventory

## Abstract

This data article is related to the research article “Comparative life cycle assessment of coffee jar lids made from biocomposites containing poly (lactic acid) and banana fiber”. The article reports the model parameters used to construct each stage and unit process inventory of the life cycle of coffee jar lids, and the subsequent inventories of the investigated system. It also contains details of calculations and descriptions of inventory uncertainties. Primary data were obtained from lab-scale and pilot-scale tests during product preparation. Secondary data collection was based on detailed review of related international and regional literature, databases and recognized web sites. The data presented here can be used by future life cycle assessment studies on natural fiber composites in packaging applications.

Specifications table

**Table:** 

Subject	Environmental Engineering
Specific subject area	Life Cycle Assessment
Type of data	Table
How data were acquired	Data related to the agricultural production of the raw materials, as well as transport to the factory, were obtained by means of direct questions to the appropriate technologist or responsible of the concerned stages. Transformation processes data were taken from characterization assays of materials and products, material and energy balances from laboratory and pilot tests. Regional reports, scientific literature, databases (Ecoinvent 3.3), personal communications with stakeholders and own calculations were also used to consolidate data.
Data format	Raw and processed.
Parameters for data collection	Representative samples selected to characterize materials, mass and energy balance of unit processes. Data collected on-site or extracted from Ecoinvent 3.3 by using the software program SimaPro 8.3 (PRe-Consultants, the Netherlands).
Description of data collection	Much of primary data for the coffee jar lids life cycle was collected directly from real processes at laboratory and pilot scale. Supplementary primary data were collected via face-to-face, telephone and email communication and interviews with stakeholders. Secondary data were generated through trustworthy site visits, technical and academic literature and regional database analysis.
Data source location	Institution: Universidad Nacional de Colombia sede Manizales City: Manizales Country: Colombia
Data accessibility	With the article
Related research article	L.J. Rodríguez, S. Fabbri, C.E. Orrego, M. Owsianiak, Comparative life cycle assessment of coffee jar lids made from biocomposites containing poly(lactic acid) and banana fiber, J. Environ. Manage., 2020, In Press [1].

## Value of the Data

• The data increase transparency of the LCA reported in the main article.
• The data can be used by other researchers or by stakeholders that are interested in modelling of natural fiber composites in packaging applications.
• The modelling parameters and the unit process inventories can be adapted to generate similar process inventory.
• The data has Latin-American relevance, and originates mainly from Colombia.

## 1 Data description

This article reports the modelling parameters and the life cycle inventory data of stages for manufacturing and landfilling of coffee jar lids made from biocomposites with banana fiber. Table 1 contains all the parameters used to calculate the inventory data for each stage of the life cycle: cultivation, transport, production and preparation of the banana pseudostem, the fabrication of the lids and end of life. These parameters were based on direct measurements from laboratory and pilot tests, by asking direct questions to producers, companies, and analysis of local literature and web data. Table 1 also shows the description of the data and how the calculations were made. Table 2, Table 3, Table 4, Table 5, Table 6, Table 7, Table 8, Table 9, Table 10 complement table 1 with data from the region of interest, namely the volumes of production taken from local government databases, soil characteristics and percentage of fertilizer emissions from studies of the region, transport distance from google maps and own calculations, fuel emissions from a Colombian database and electrical demand of the machines based on information from a local company and laboratory data. The tables 11–20 refer to all input and outputs flows a functional unit of 1 coffee jar lid for each process throughout the biochar life cycles constructed using model parameters given in tables 1–10. These tables include data-related uncertainties following the ecoinvent pedigree approach and the squared geometric standard deviation.

**Table 1:** Model parameters and data sources for foreground processes in the lid life cycle. Sensitivity scenario was treated BF with economic allocation between banana fruit and fiber. Biocomposite composition: 40% BF, 30% HDPE and 30% PLA. LCI data source “Banana {CO}|banana production| Alloc Def, U” was improved when required according local information and conditions.

Parameter		Unit	Note	Source
Agricultural production			Technified farming system was selected. Economic allocation factor for banana fiber was 8.3%.	
Inputs				
Land use	0.38	m^2^/kg/year	Estimated from measured production of banana fiber from technified banana farm. See technified cultivation table 2. Average yield = 342 kg/ha.	Calculated^2^
Photosynthesis	26	μmol CO_2_m^−1^ s^−1^	The carbon dioxide from the air and solar energy for the photosynthesis process presents maximum photosynthesis rates.	[2]
Carbon dioxide			26 CO_2_ [μmol CO_2_m^−1^ s^−1^] * 44 Molecular weight CO_2_[g/mol]= CO_2_ [μg/ m^−1^ s^−1^]. Then is calculated CO_2_ [kg/m*year].	
Fertilizers				
Urea	462	kg/ha/year	The amount of fertilizers used are according to the soil studies include pH, and the content of organic matter, phosphorus, sulfur, iron, magnesium, zinc, copper and boron. These considerations depend not only on the crop under consideration but also on the climatic conditions of the soil. Table 3 show the soil characteristics of the region.	Interview/ata^3^
DAP Phosphate	152	kg/ha/year		
Potassium chloride	692	kg/ha/year		
Organic matter	4000	kg/ha/year		
CAL	875	kg/ha/year		
Maintenance				
Prune weeds	4.80	m^2^/kg/year	The maintenance is carried out by pruning 5-7 times/year. The extraction process is done by a scythe 1.07 kw fuel machine, weight of 7.5 kg, rate 1.5l/h fuel and 0.05l oil/fuel. It is estimated 1ha/day and lifetime of 10 years.	Calculated^2^ Interview/data^3^
Glifosato	1.56	kg/ha/year	Although, pests and diseases can be prevented with manual maintenance practices and other insects. Pesticides/ herbicides are used in necessary cases and the ultimate goal is to reduce their dependency completely. In this case they are considered some of the most used.	[3]
Mancozeb	6.87	kg/ha/year		[3]
Chlothaonil	0.41	kg/ha/year		[3]
Output				
Emissions				
Amonia (air)	22.06	%	The amount of nitrogen was calculated based on the fertilizers applied. A proportion of nitrogen is evaporated as ammoniac NH_3_ to the air. Average losses of different regions were taken to perform the calculation, see Table 4	Calculated^2^
Phosphorous (water)	13.00	%	The amount of phosphorus was calculated based on the fertilizers applied. Losses of phosphorus are emitted to water. Average losses of different regions were taken to perform the calculation, see Table 4	Calculated^2^
Potassium (water)	34.33	%	The amount of potassium was calculated based on the fertilizers applied. Losses of potassium are emitted to water. Average losses of different regions were taken to perform the calculation, see Table 4	Calculated^2^
Calcium (water)	32.64	%	The amount of calcium was calculated based on the fertilizers applied. Losses of calcium are emitted to 60% to soil and 40% to water. Average losses of different regions were taken to perform the calculation, see Table 4	Calculated^2^
Magnesium (water)	1.00	%	The amount of magnesium was calculated based on the fertilizers applied. Losses of magnesium are emitted to 60% to soil and 40% to water. Average losses of different regions were taken to perform the calculation, see Table 4	Calculated^2^
Postharvest residues Organic waste	27.92	ton/ha/year	92% correspond to water and 8% to solids (determined using a Shimadzu Moisture Balance MOC-120H). Table 5 shows the composition of residues of banana fiber.	Measured^1^
Transport 1				
Distance	0.12	tkm	Distance corresponds to transport the pseudostem from the farms to the collecting centers of the subregions (T_1_). The distance T_1_ was estimated by the center of gravity method. Data was calculated according to total production of pseudostem 157940 ton/year, car capacity of 1.5 tons and average distance from Table 6.	Calculated^2^
Natural gas	0.06	m^3^/km	The transportation of the farms corresponds to a small car of 1.4 L which has sufficient capacity for tertiary roads. Selected car of 1.5 tons capacity, is estimated to consume 10 m^3^/160 km.	[4]
Emissions				
Carbon dioxide, fossil	Table 7	kg/m^3^	The emissions are similar to a large passenger car. The CO_2_ and CH_4_ emissions from transportation was calculated based in the Mining Energy Planning Unit, Colombia's energy emissions calculator. These values have been considered and used to modify some of the values of the Ecoinvent databases.	[5]
Methane	Table 7	kg/m^3^		[5]
Fiber production				
Rate production	20	kg/h	The desfibrating process is done by a 10 HP fiber decorticator diesel machine, weight of 125 kg, rate production was 20kg/h and lifetime of 10 years (see Fig.1). A machine operation by diesel was selected from Ecoinvent and data were recalculated.	Measured^1^
Diesel	1.03,E-02	l/kg*	This data was calculated based on 70 kg of wet* banana fiber extracted.	Measured^1^
Emission				
CO_2_	Table 7	kg/m^3^	The CO_2_ emissions of diesel consume was calculated based in the Mining Energy Planning Unit, Colombia's energy emissions calculator. These values have been considered and used to modify some of the values of the Ecoinvent databases.	[5]
Washing and Drying	5.00E-3	m^3^/kg*	After extraction, the fibers are submerged in water for 24 hours. wet* banana fiber	Measured^1^
Transport 2				
Dry BF	0.08	Tkm	Distance corresponds to transport the BF from the sub regions to plant located in Manizales (T_2_). The distance was estimated from google maps. Data was calculated according to total potential production of fiber Table 2, average distances from Table 6 and a truck capacity of 3 tons.	Calculated^2^
Diesel	0.24	gal/km	The transportation T_2_ corresponds to a truck with a capacity of 3 tons, which is estimated to consume 24 gal/100km of diesel.	Calculated^2^
Emissions				
CO_2_	Table 7	kg/m^3^	The CO_2_ emissions from transportation was calculated based in the Mining Energy Planning Unit, Colombia's energy emissions calculator. These values have been considered and used to modify some of the values of the Ecoinvent databases.	[5]
Fiber preparation				
Rate milling production	Table 8	kg/h	Based on selected machine, the data of a selected ecoinvent machine was recalculated. Lifetime 20 years.	Calculated^2^
Milling electricity demand	Table 8	kWh	The banana fiber were conditioned by the grinding process. The demand of electricity was calculated based on the performance of the machine at the laboratory level to two industrial machines.	Calculated^2^
Pretreatment				
Anhydride Acetic	10.80	ml/kg	The chemical reagents (AA, EP and blends of AA and EP, AA_EP) were dissolved in acetone (chemical to acetone weight ratio, 1:10; AA to EP weight ratio in AA_EP, 1:1). Raw fibers (raw fiber to chemical weight ratio, 1:20) were immersed in the chemical/acetone solution for 24 h at 20 C. The fibers were then washed several times in sufficient amounts of acetone and distilled water to ensure the removal of all reagent residues. A glass container is required, approx. 2kg and lifetime 5 years	Calculated^2^
Epiclorohydrine	18.30	ml/kg		Calculated^2^
Acetone	71.19	ml/kg		Calculated^2^
Water	10	l/kg		Calculated^2^
Rate drying machine	Table 8	Kg/h	Based on selected machine, the data of a selected ecoinvent machine was recalculated. Lifetime 20 years.	Calculated^2^
Drying electricity demand	Table 8	kWh	Due to the hydroscopic characteristic of natural fibers, before blend with the other hydrophobic materials to reduce problems during extrusion process, therefore, the BPF were dried in an oven at 60°C for 24 h. The demand of electricity was calculated based on the performance of the machine at the laboratory level to scale it to industrial machines.	Calculated^2^
Lid production				
HDPE	Table 9	g/lid	The annual demand is about 600000 units or 8 tons of material, therefore, between 0.8 and 3.2 tons of fiber by year in biocomposites of 10% and 40% of fiber. Based on 1 lid requires 13.44 g material. For different blends please see table 10	Calculated^2^
PLA	Table 9	g/lid		Calculated^2^
PE-g-MA	1.12	g/lid	Correspond to 8% of the BF/HDPE/PLA total weigh	Calculated^2^
Extrusion electricity demand	Table 8 and 10	kWh	The 10 blends were made in a torque rheometer as experimental process, with a 98% of efficiency of material. For industrial process was recommended a counterrotating twin-screw extruder with a diameter 71 mm, rpm 600, L/D 32-54, Motor kw: 132 and Torque NM: 1050. The demand of electricity was calculated based on the performance of the biocomposite at the laboratory level to scale it to industrial machines.	Calculated^2^
Injection molding electricity demand	Table 8 and 10	kWh	The injection molding machine has 300 tons of pressure in the cavity, demand of 7 kWh and produce 8 lids per mold every 12s of 13.44 g of weight.	Calculated^2^
Transport to landfill				
Lid	6.7E-5	Tkm	Distance corresponds to transport the disposable lids post consume from Manizales to landfill. The distance was estimated from google maps. Data was calculated according to annual demand of lids is about 600000 units or 8 tons of material and truck capacity of 10 tons.	Calculated^2^
Emissions				
CO_2_	Table 7	kg/m^3^	The CO_2_ emissions from transportation was calculated based in the Mining Energy Planning Unit, Colombia's energy emissions calculator. These values have been considered and used to modify some of the values of the Ecoinvent databases.	[5]
Landfill	1	Lid	For Discharging 1 lid, two disposals were considered, according to amount of HDPE as synthetic plastic and PLA and BF as biodegradable materials.	
HDPE	5.37	Gr	Landfill of plastic waste	Ecoinvent
PLA and BF	8.07	Gr	Landfill of biodegradable waste	Ecoinvent

1 Average output of a set of standard experimental assays at lab-scale or pilot-scale.

2 Data were mathematically determined from experimental work or secondary data from reputable sources.

3 Agrosavia, Corporación Colombiana de investigación agropecuaria, 2018 Comité de cafeteros de Caldas-Manizales, 2018, and Gobernación-Caldas, 2018, Alcadía-Manizales, 2018.

**Table 2:** Banana fruit and banana fiber production volume for two types of cultivation (non-technified and technified) within the selected region used for the allocation at farming stage.

Agricultural production	Area (Ha)	Banana fruit (ton)	Fiber (ton)	Rate (%)	Banana (kg/ha)	Fiber (kg/ha)	Banana US$ millions^1^	Fiber US$ millions^2^	Source
Non-Technified	21359	235216	3058	90.26	11012.35	143.17	70.56	6.47	Interview/data^3^ and Calculated^4^
Technified	967	25390	330	9.74	26270.05	341.51	7.62	0,70	
TOTAL	22326	260606	3388		12499.48	162.50	78.18	7.17	

1 US$ 0.3/kg banana.

2 US$ 2.1/kg fiber (UVR 3000 December 2018).

3 Data from various regional institutions (2018): Agrosavia, Corporación Colombiana de investigación agropecuaria; Comité de cafeteros de Caldas, Manizales; Gobernación-Caldas; Alcadía-Manizales.

4 The data was mathematically determined based on experimental measurements, or from secondary data such as literature.

**Table 3:** Soil characteristics of the studied region.

	pH	Organic matter	Phosphorus	Sulfur	Iron	Magnesium	Zinc	Copper	Boron	Source
Min	4.85	1.73	4.03	0.07	32.59	0.39	0.45	0.36	0.07	Interview/ data^1^
Max	6.38	5.57	51.71	16.88	328.96	18.81	8.48	10.95	1.88	

1 Agrosavia, Corporación Colombiana de investigación agropecuaria, and Comité de cafeteros de Caldas-Manizales, 2018.

**Table 4:** Gaseous emissions from fertilizer components.

Regions	1	2	3	4	5	6	7	Unit	Source
Ammonia	7.5	1.0	24.7	33.0	70.0	0.7	32.0	%	[6,7]
Phosphorous	0	27.0	43.0	2.0	9.0	10.0	0	%	[6]
Potassium	25.0	65.0	22.5	36.0	-	-	-	%	[6]
Calcium	70.0	7.7	-	-	-	-	-	%	[6]
Magnesium	-	1.0	-	-	-	-	-	%	[6]

**Table 5:** Composition of product and by-product.

	Unit	Pseudostem	Organic waste	Measured through proximate analysis
Ash (DB)^1^	%	9.34	27.43	Calcination at 600°C
Moisture	%	12.45	11.09	Moisture analyzer
K	%	2.95	10.31	Flame atomic absorption
Ca	%	1.47	0.46	Flame atomic absorption
Mg	mg/Kg	1060.58	233.42	Flame atomic absorption
P	mg P/L	473.53	447.00	Stannous chloride
Fat (DB)^1^	%	0.64	0.92	Soxhlet
Protein (DB)^1^	%	0.50	1.06	Kjeldhal
Fiber (DB)^1^	%	14.95	5.26	Gravimetric
Carbohydrates (DB)^1^	%	74.60	65.33	Nitrogen-free extract

1 DB (dry base).

**Table 6:** Distance to transport pseudostem from farms to collecting center of the subregions (T_1_). Distance to transport banana fiber from collecting center to manufacturing plant (T_2_).

Sub region	T_1_^1^(km)	T_2_^2^(km)	
Magdalena	124	136.0	
High east	96	108.0	
North	71	51.0	
South central	80	0	
High West	95	81.2	
Low West	50	55.1	

1 All distances are average values between farm and gathering center, mathematically determined from measured data, by using the center of gravity method.

2 All distances were calculated by distance between biomass collecting point and the plant, calculated using Google maps, the data was mathematically determined based on measured data, or secondary data such as literature.

**Table 7:** Emission factors for fuels.

Emission Species	CO_2_	CH_4_	Unit
Natural Gas	1.9800	0.0033	kg/m^3^
Diesel	0.2837		kg/m^3^

Data Source, Mine and Energy Planning Unit, Colombia's [5].

**Table 8:** Equipment specifications for banana fiber preparation

Process	Consumption	Unit	Rate	Unit	Source
Milling	265.50	kWh	8800	kg/h	[8, 9]
Dry	0.06	kWh/kg	160	kg/h	[10]
Extrusion	0.25	kWh/kg	250	kg/h	[11]
Injection	7.00	kWh	300000	kg/h	Interview – Local company

**Table 9:** Amount of banana fiber, HDPE and PLA per 1 lid.

	Blend (%) BF HDPE PLA			1 Lid (12.9 g) BF HDPE PLA		
1	10	45	45	1.3	5.8	5.8
2	20	40	40	2.7	5.1	5.1
3	30	35	35	3.9	4.5	4.5
4	40	30	30	5.1	3.9	3.9
5	40	60	0	5.1	7.8	0
6	40	0	60	5.1	0	7.8

Data was mathematically determined based on measured data, or secondary data such as literature.

**Table 10:** Experimental electricity demand for extrusion and injection.

	Blend (%) BF HDPE PLA			Torque Rheometer	Extrusion	Injection	Unit
1	10	45	45	0.072	7.30 E-04	6.01E-04	kWh/kg
2	20	40	40	0.091	9.19 E-04	7.57E-04	kWh/kg
3	30	35	35	0.102	10.27E-04	8.46E-04	kWh/kg
4	40	30	30	0.098	9.98 E-04	8.21E-04	kWh/kg
5	40	60	0	0.078	7.89 E-04	6.49E-04	kWh/kg
6	40	0	60	0.084	8.53 E-04	7.03E-04	kWh/kg

Data measured during the real process and using an instrument.

**Table 11:** Inventory for the unit process of cultivation stage, output 46.62 kg pseudostem to produce 1 kg (dry banana fiber). The unit processes are representative of the farming systems in Colombian selected region. “Banana {CO}|banana production| Alloc Def, U” was the LCI data source that was modified according to model parameters of the regional conditions in Table 1. Some data were changed such as occupation land, fertilization, maintenance and emissions. Technified process including irrigation and tractor use was removed and remained data were recalculated from ecoinvent.

Activity	Amount	Unit	Pedigree	σ_g_^2^	Source
Product					
Output pseudostem	46.62	kg			For 1 lid is required 0.297 kg
Resources					
Occupation, permanent crop, irrigated	3.16E-02	m^2^a	(1,1,1,1,1,1.1)	1.1	See Table 1
Transformation, from permanent crop, irrigated	1.49E-03	m^2^	(1.1,1.05,1.03,1.001,1,2.2)	1.238	Calculated based on Ecoinvent
Transformation, to permanent crop, irrigated	1.49E-03	m^2^	(1.1,1.05,1.03,1.001,1,2.2)	1.238	Calculated based on Ecoinvent
Carbon dioxide, in air	4.15E-03	kg	(1.05,1,1.1,1,1,na)	1.113	See Table 1
Energy, gross calorific value, in biomass	4.49E-02	MJ	(1.1,1.05,1.03,1.001,1,na)	1,117	Ecoinvent
Materials/fuels					
Establishing orchard {GLO}| market for establishing	2.07E-05		(1.1,1.05,1.03,1.001,1,na)	1.117	Calculated based on Ecoinvent
Agricultural machinery, unspecified {RoW}| production | Alloc Def, U	3.32E-05		(1.1,1.05,1.03,1.001,1,na)	1.117	Calculated based on Ecoinvent
Agricultural machinery, tillage {RoW}| production | Alloc Def, U	3.32E-05		(1.1,1.05,1.03,1.001,1,na)	1.117	Calculated based on Ecoinvent
Urea, as N {GLO}| market for | Alloc Def, U	1.49E-03	kg	(1,1,1,1,1,na)	1	See Table 1
Phosphate fertiliser, as P2O5 {GLO}| market for | Alloc Def, U	4.98E-04	kg	(1,1,1,1,1,na)	1	See Table 1
Potassium chloride, as K2O {RoW}| potassium chloride production | Alloc Def, U	2.16E-03	kg	(1,1,1,1,1,na)	1	See Table 1
Compost {RoW}| treatment of biowaste, composting | Alloc Rec, U	1.26E-02	kg	(1,1,1,1,1,na)	1	See Table 1
Soil pH raising agent, as CaCO3 {GLO}| market for | Alloc Def, U	2.74E-03	kg	(1,1,1,1,1,na)	1	See Table 1
Mowing, by motor mower {RoW}| processing | Alloc Def, U	1.06E-05	m^3^	(1,1,1,1,1,na)	1	See Table 1
Packaging, for fertilizers or pesticides {GLO}| market for packaging, for fertilizers or pesticides | Alloc Def, U	3.23E-04	kg	(1.1,1.1,1.03,1.001,1,na)	1.148	Calculated based on Ecoinvent
Land use change, perennial crop {CO}| market for land use change, perennial crop | Alloc Def, U	1.67E-06	ha	(1.1,1.1,1.03,1.001,1,1.2)	1.238	Calculated based on Ecoinvent
Packaging film, low density polyethylene {GLO}| market for | Alloc Def, U	1.04E-05	kg	(1.1,1.1,1.03,1.001,1,na)	1.148	Calculated based on Ecoinvent
Glyphosate {GLO}| market for | Alloc Def, U	8.30E-06	kg	(1,1,1.1,1,1,na)	1.1	See Table 1
Mancozeb {GLO}| market for | Alloc Def, U	2.16E-05	kg	(1,1,1.1,1,1,na)	1.1	See Table 1
Chlorothalonil {GLO}| market for | Alloc Def, U	1.66E-06	kg	(1,1,1.1,1,1,na)	1.1	See Table 1
Fruit tree seedling, for planting {GLO}| market for fruit tree seedling, for planting | Alloc Def, U	9.88E-07	p	(1.1,1.1,1.03,1.001,1,na)	1.148	Calculated based on Ecoinvent
Planting tree {GLO}| market for planting tree | Alloc Def, U	6.30E-09	p	(1.1,1.1,1.03,1.001,1,na)	1.148	Calculated based on Ecoinvent
Emissions to air					
Ammonia	2.05E-06	kg	(1,1,1.1,1,1,1.2)	1.228	See Table 1
Nitrogen oxides	2.51E-06	kg	(1.1,1.2,1.03,1.001,1,1.4)	1.485	Calculated based on Ecoinvent
Dinitrogen monoxide	3.48E-06	kg	(1.1,1.2,1.03,1.001,1,1.4)	1.485	Calculated based on Ecoinvent
Water/m3	6.02E-03	M3	(1.1,1.2,1.03,1.001,1,na)	1.148	Calculated based on Ecoinvent
Emissions to water					
Phosphorous (river)	2.76E-03	kg	(1,1,1.03,1,1,1.5)	1.502	See Table 1
Potassium (river)	3.51E-07	kg	(1,1,1.03,1,1,1.5)	1.502	See Table 1
Calcium (river)	1.12E-07	kg	(1,1,1.03,1,1,1.5)	1.502	See Table 1
Magnesium (river)	3.44E-09	kg	(1,1,1,1,1,1.5)	1.50	See Table 1
Water, CO (river)	1.31E-03	m^3^	(1,1,1,1,1,na)	1	25% of water of organic waste, see Table 1.
Water, CO (groundwater)	3.93E-03	m^3^	(1,1,1,1,1,na)	1	75% of water of organic waste, see Table 1.
Emissions to soil					
Calcium (agricultural)	1.66E-07	kg	(1,1,1.03,1,1,na)	1.03	see Table 1
Magnesium (agricultural)	5.15E-09	kg	(1,1,1.03,1,1,na)	1.03	see Table 1
Waste to treatment					
Postharvest residues Banana	4.63E-01	kg	(1,1,1,1,1,na)	1	see Table 1 and Table 5

**Table 12:** Inventory for the pseudostem transport stage from farm to collecting center, output 1 km. The data source “Transport, passenger car, large size, natural gas, EURO 5 RoW| transport, passenger car, large size, natural gas, EURO 5 | Alloc Def, U” was selected due to emissions similar to those of current transport.

Activity	Amount	Unit	Pedigree	σ_g_^2^	Source
Product					
Transport 1	**1**	**km**			For 1 Lid is required 1.9,E-03 km
Materials/fuels					
Natural gas, high pressure {RoW}| market for | Alloc Def, U	6.25E-02	m^3^	(1.1,1,1,1,1,2)	2	See Table 1
Emissions to air					
Carbon dioxide, fossil	1.24E-06	kg	(1,1,1,1,1,1.05)	1.05	See Table 7
Methane	2.05E-09	kg	(1,1,1,1,1,1.5)	1.5	See Table 7

**Table 13:** Inventory for the decortication unit process, output 1 h of wet banana fiber. Process similar to “machine operation, diesel, < 18.64 kW, generators GLO”.

Activity	Amount	Unit	Pedigree	σ_g_^2^	Source
Product					
Output wet Banana Fiber decorticated	**1**	**h**			For 1 lid is required 1.15 sec
Materials/fuels					
Diesel {RoW}| market for | Alloc Def, U	4.82E-02	kg	(1.1,1,1,1,1,2)	2.003	See Table 1 diesel density 832kg/m3
Emissions to air					
Carbon dioxide	1.54E-00	kg	(1,1,1,1,1,1.1)	1.05	See Table 1 and Table 7
Emissions to water					
Water, CO	4.10E-02	m^3^	(1,1,1,1,1,na)	1	Calculated 88% of pseudostem is water

**Table 14:** Inventory for the washing and drying unit processes, output 1 kg banana fiber.

Activity	Amount	Unit	Pedigree	σ_g_^2^	Source
Product					
Output Banana Fiber washed and Dried	**1**	**kg**			For 1 lid, BF is 6.38E-03 kg
Resources					
Water, river, CO	2.80E-02	m^3^	(1,1,1,1,1,na)	1	See Table 1
Emissions to air					
Water/m3	4.32E-03	m^3^	(1,1,1,1,1,na)	1	Calculated 82% of wet fiber is water
Emissions to water					
Water, CO	2.80E-03	m^3^	(1,1,1,1,1,na)	1	Calculated 10% of used water is discarded

**Table 15:** Inventory for banana fiber transport from collecting center to transformation company, output 1 tkm. "Transport, light commercial truck, diesel powered, Southeast/tkm/RNA".

Activity	Amount	Unit	Pedigree	σ_g_^2^	Source
Product					
Transport 2	1	tkm			For lid is required 5.48,E-04 km
Materials/fuels					
Diesel, at refinery/l/US	0.26	l	(1.1,1,1,1,1,2)	2.003	See Table 1
Emissions to air					
Carbon dioxide, fossil	70.03E-05	kg	(1,1,1,1,1,1.1)	1.05	See Table 7

**Table 16:** Inventory for the milling unit process, 1 kg of banana fiber.

Activity	Amount	Unit	Pedigree	σ_g_^2^	Source
Product					
Output Banana Fiber Milled	**1**	**kg**			For 1 Lid is required 5.80,E-03 of BF
Electricity/heat					
Electricity, low voltage {BR}| market for | Alloc Def, U	3.25E-02	kWh	(1.1,1.1,1,1.02,1.2,na)	1.256	See Table 1 and 8
Chipper, stationary, electric {GLO}| market for | Alloc Def, U	1.48E-06	p	(1.2,1.1,1,1.001,1.2,na)	1.316	See Table 1 and 8

**Table 17:** Inventory for the unit process scenarios for chemically treated banana fiber, output 1 kg.

Activity	Amount	Unit	Pedigree	σ_g_^2^	Source
Product					
Output Banana Fiber treated	**1**	**kg**			For 1 Lid is required 5.80E-03 of BF
Resources					
Water, well, in ground, CO	2.16E-03	m^3^	(1,1,1,1,1.2,na)	1.2	Measured
Acetic anhydride {RoW}| market for | Alloc Def, U	1.00E-02	kg	(1,1,1,1,1.2,na)	1.2	Calculated using data in Table 1 and Table 9. Acetic anhydride density 1.08 g/cm³
Epichlorohydrin {GLO}| market for | Alloc Def, U	9.00E-03	kg	(1,1,1,1,1.2,na)	1.2	Calculated using data in Table 1 and Table 9. Epichlorohydrin density 1.18 g/cm³
Acetone, liquid {GLO}| market for | Alloc Def, U	9.00E-02	kg	(1,1,1,1,1.2,na)	1.2	Calculated using data in Table 1 and Table 9. Acetone density 784 kg/m³
Packaging glass, white {GLO}| market for | Alloc Def, U	1.70E-03	kg	(1,1,1,1,1.2,na)	1.2	Calculated
Emissions to air					
Water/m3	2.14E-04	m^3^	(1,1,1,1,1.2,na)	1.2	Measured
Waste to treatment					
Wastewater, average {Europe without Switzerland}| treatment of wastewater, average, capacity 1E9l/year | Alloc Def, U	1.93E-03	m^3^	(1.2,1.1,1.1,1.1,2,na)	2.261	Measured

**Table 18:** Inventory for the drying unit process, 1 kg of banana fiber.

Activity	Amount	Unit	Pedigree	σ_g_^2^	Source
Product					
Output Banana Fiber Dried	**1**	**kg**			For 1 Lid is required 5.20E-03 of BF
Electricity, low voltage {BR}| market for | Alloc Def, S	6.00E-02	kWh	(1.1,1.1,1,1.02,1.2,na)	1.256	See Table 1 and 8
Technical wood drying facility {RoW}| construction | Alloc Def, U	1.87E-07	p	(1.2,1.1,1,1.001,1.2,na)	1.316	See Table 1 and 8
Emissions to air					
Water/m3	9.33E-05	m^3^	(1,1,1,1,1,na)	1	Measured

**Table 19:** Inventory for the extrusion unit process, 1 kg of biocomposite. Scenario: 40% banana fiber, 30% HDPE and 30% PLA. Production by extrusion and thermoforming of plastic sheets. {CA-QC}| production | Alloc Def, U and Injection molding {CA-QC}|, injection molding | Alloc Def, U.

Activity	Amount	Unit	Pedigree	σ_g_^2^	Source
Product					
Production of lids	**1**	**lid**			Blend 40% BF 30% HDPE and 30% PLA
Extrusion					
Materials					
Polyethylene, high density, granulate {GLO}| market for | Alloc Def, U	3.90E-03	kg	(1,1,1,1,1,na)	1	Ecoinvent see Table 1
Polylactide, granulate {GLO}| market for | Alloc Def, U	3.90E-03	kg	(1,1,1,1,1,na)	1	Ecoinvent see Table 1
Maleic anhydride | market for | Alloc Def, U	1.12E-03	kg	(1,1,1,1,1,na)	1	Ecoinvent see Table 1
Banana Fiber	5.10E-03	kg	(1,1,1,1,1,na)	1	Ecoinvent see Table 1
Electricity/heat					
Electricity, low voltage {BR}| market for | Alloc Def, U	9.98E-04	kWh	(1.1,1,1,1.02,1.2,na)	1.21	See Table 8 and 10
Injection molding					
Electricity/heat					
Electricity, low voltage {BR}| market for | Alloc Def, U	8.21E-04	kWh	(1.1,1,1,1.02,1.2,na)	1.21	See Table 8 and 10

**Table 20:** Inventory for the transport stage of disposable lids to landfill, output 1 km. “Transport, truck 10-20t, EURO5, 100%LF, empty return/GLO Mass”.

Activity	Amount	Unit	Pedigree	σ_g_^2^	Source
Transport to landfill	1	tkm			For 1 Lid is required 6.7E-05
Materials/fuels					
Diesel, from crude oil, consumption mix, at refinery, 200 ppm sulphur EU-15 S System - Copied from ELCD	5.98E-02	kg	(1.1,1,1,1,1,2)	2.003	Ecoinvent
Emissions to air					
Carbon dioxide, fossil	1.93E-06	kg	(1,1,1,1,1,1.1)	1.05	See Table 7

## 2 Experimental design, materials, and methods

The parameters and inventory data of coffee jar lids were generated in three stages, cultivation of banana, fiber and lid production. Data on the cultivation stage were collected from local government and regional literature. Data on fiber production were obtained from a pilot process using approximately 1.8 tons of pseudostem collected from three farms. The banana fiber was extracted by a fiber decorticator, washed and dried. Data regarding material flows were measured with an industrial balance. Chemical components of fiber and residue were measured through proximate analysis. Transport distances between locations of the different life cycle stages were taken from Google maps and the fuel emissions from regionalized inventories [2]. The lid production was conducted at laboratory scale, the fibers were milled and chemically treated. Mass balance and time were taken and calculated. Six blends of Poly (lactic acid), PLA (0 – 60%), High Density Polyethylene, HDPE (0 – 60%), and Banana Fiber, BF (10-40%) were made in a torque rheometer as experimental process and then injected. Data on mass, energy and machine characteristics were recorded and used to calculate data for industrial machines.

## 3 Unit processes and LCI data

The information given here includes all input and outputs flows from each process throughout the biochar life cycles constructed using model parameters given in Section 3. Pedigree criteria and subsequent geometric standard deviations squared (σ _g_^2^) underlying uncertainty analysis were described in detail in Rodríguez et al. (2020) [1].
